# Reduced use of antimicrobials after vaccination of pigs against porcine proliferative enteropathy in a Danish SPF herd

**DOI:** 10.1186/1751-0147-51-1

**Published:** 2009-01-07

**Authors:** Hanne Bak, Poul Henning Rathkjen

**Affiliations:** 1 Boehringer-Ingelheim Vetmedica, Strødamvej 52, DK-2100 Copenhagen Ø, Denmark

## Abstract

The present study explored whether the use of group medication with antibiotics in a Danish pig herd was reduced after vaccination of the pigs against proliferative enteropathy (PE) caused by *Lawsonia intracellularis*. 7900 pigs originating from a single commercial sow herd were vaccinated against *L. intracellularis*, whereas 7756 pigs were kept as non-vaccinated controls. The pigs were included batch-wise in the study with every second batch being vaccinated. In the vaccinated batches, the consumption of oxytetracykline to treat PE was reduced by 79%, with a significantly lower number of pigs being treated (*P *< 0.0001). Vaccination also resulted in a highly significant improvement of average daily weight gain (+ 46 g/day; *P *= 9.55 × 10^-31^) and carcase weight (+ 1.25 kg; *P *= 4.54 × 10^-05^) as well as a shortened fattening period (-8 days; *P *= 2.01 × 10^-45^).

## Findings

In pig producing countries,*Lawsonia intracellularis *is a common intestinal infection with severe economic consequences due to decreased growth rates and feed conversion. In many herds, continuous therapy with antibiotics is required to control the consequences of the infection [1]. Therefore, *L. intracellularis *lead to increased use of antibiotics. In Denmark, 93% of the pig herds are infected with *L. intracellularis *[2]. Danish farmers use a low amount of antibiotics compared to farmers in many other pig producing countries [3], but Danish farmers experience continuous pressure from the public to reduce the amount of antibiotics further. Most of the antibiotics for Danish pigs are used for weaners, and the most frequently used compounds are tetracykline and macrolides [4], both useful for treatment of PE. Hence, a significant reduction of the use of antibiotics in the Danish pig production might be achieved by an alternative approach to PE.

An obvious alternative to antibiotics is vaccination. Vaccination against *L. intracellularis *has proven effective in controlling PE and improving production parameters in infected herds [5]. Vaccination has also resulted in a significant reduction in the amount of antibiotics used [6] or has completely replaced the antibiotics [7]. However, these studies were carried out in populations treated with antibiotics at a higher level than generally done in Denmark. The question is whether vaccination against PE can reduce the already low amount of antibiotics used for Danish pigs further. The present study evaluates the effect of vaccination against PE on the use of antibiotics and selected production parameters in a herd with limited use of antibiotics.

The study was carried out in a 650-sow specific pathogen free (SPF) herd with a level of *Salmonella *infection below the detection level. The herd produced one batch of approximately 1000 pigs every 3rd week. At 31 days of age, the pigs were weaned and distributed between 2 nursery sites, and at 30 kg live weight, half of the pigs in a batch were moved to two fattening sites on the same farm, whereas the other half was sold to another farmer for fattening. Before the study started, the farmer signed an informed content sheet describing the trial. The experiment was approved by the Danish Medicines Agency (Journal No. 2615-114). The herd used no prophylactic medications. Group medication of pigs was necessary to treat weaning diarrhoea 1–2 weeks after weaning in half of the batches, and a second treatment for diarrhoea was used for approximately every third batch to treat diarrhoea in older pigs. This second round of diarrhoea was caused by *L. intracellularis*. Infection with *L. intracellularis *was confirmed by laboratory examination of diseased intestines, and no other intestinal pathogens could be isolated from these intestines. A serological analysis by ELISA [8] was executed 5 weeks after the proposed age for vaccination to confirm the correctness of timing of vaccination. The presence of *L. intracellularis *was surveyed throughout the study by analyses of *L. intracellularis *antibodies in blood by ELISA.

**Figure 1:**
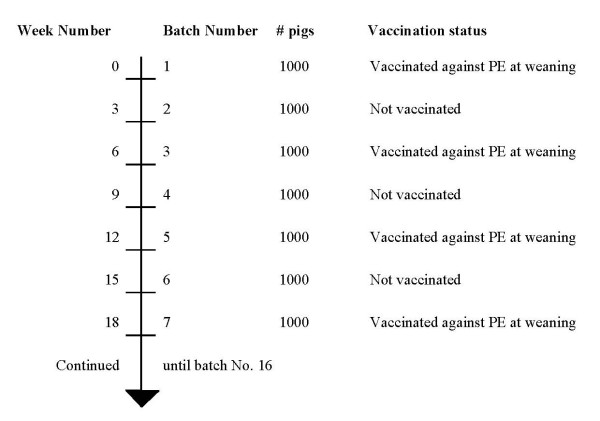
**Study design**. Principle of inclusion of pigs in a study based on a modified parallel group design from a herd, which weaned 1000 pigs every 3^rd ^week. The study compares pigs vaccinated against proliferative enteropathy (PE) caused by *Lawsonia intracellularis *with non-vaccinated pigs.

The study was performed in a modified version of a parallel group design with consecutive, batchwise inclusion of pigs. The study included 16 batches of pigs and every second batch was vaccinated with a live oral *L. intracellularis *vaccine (Enterisol^® ^Ileitis Vet., Boehringer-Ingelheim AS, Copenhagen, Denmark), whereas remaining batches were kept as non-vaccinated controls (Figure 1). The vaccine was administered at 31 days of age in drinking water.

Before weaning, all pigs were ear tagged with an earmark representing the batch number. On the batch level, date of entrance and date of exit were recorded by the farmer. The pigs were tattooed with a specific delivery number corresponding to the earmark representing the batch before they were shipped for slaughter. For each pig, the delivery number, date of slaughter and carcase weight was collected from the slaughterhouse database.

During the trial, group medications with antibiotics were recorded. The farmer treated diarrhoea following the usual guidelines from the local veterinarian. These guidelines included criteria for initiating a treatment, i.e. clinical signs. Diarrhoea occurring 1–3 weeks after weaning was treated as weaning diarrhoea, whereas diarrhoea occurring later was treated as PE. The use of antibiotics was compared for each pharmacologic group of antibiotics. The parameters were: Batch treated yes/no and Pig treated yes/no, and results were simple counts of yes and no's. A chi-square test was carried out to compare the vaccinated and the non-vaccinated batches and pigs. The level of significance was *P *= 0.05. For both vaccinated and non-vaccinated batches, the amount of antibiotics used (gram or ml) was registered for each compound.

Production performance was compared at the individual pig level for the parameters: days from weaning to slaughter, carcase weight and average daily weight gain (ADWG). The carcase weight was obtained directly from the slaughterhouse database, whereas the days from weaning to slaughter and the ADWG for each pig was calculated from the day of weaning represented by the batch number, the date of delivery, the mean weight at weaning and the carcase weight. The data set was checked for normality with Shapiro-Wilk test and Kolmogorov-Smirnov test and statistical comparison of vaccinated pigs and non-vaccinated controls was done with Student T-tests using a significance level of *P *= 0.01.

In the nurseries, three products were used for group medication during the study: Oxytetracykline (Premedox, Virbac, Kolding, Denmark) for treatment of PE, and aminoglycoside (Apralan, Elanco Animal Health, Lyngby, Denmark) or sulpha/TMP (Trimazin, Scanvet, Fredensborg, Denmark) for treatment of post weaning diarrhoea caused by *Eschericia coli*.

Treatment for PE was administered to 4 batches: one vaccinated batch and 3 non-vaccinated batches. In the medicated vaccinated batch, only some of the pigs (pens) were treated, whereas the treatment in the 3 non-vaccinated batches was given to around 2900 out of the 3000 pigs. A statistically significant difference was observed for the number of treated pigs, both when each nursery site was analysed separately and when nursery data was merged (Table 1). The mean amount of oxytetracykline used per batch was 1041 g for the vaccinated batches and 4860 g for the non-vaccinated batches. In total, vaccination reduced the amount of oxytetracykline used for the 8 vaccinated batches by more than 30 kg.

**Table 1:** Use of oxytetracykline in 8 batches of pigs vaccinated against proliferative enteropathy (PE) caused by *Lawsonia intracellularis *compared to 8 non-vaccinated batches.

	**Vaccinated**	**Non-vaccinated**	**Reduction after vaccination**	***P*-value^a^**
Total # pigs	7900	7756	-	-

# batches	8	8	-	-

# batches treated against PE	1	3	2	0.2482

# pigs treated against PE Nursery site 1	300	2346	2046	< 0.0001

# pigs treated against PE Nursery site 2	220	499	279	< 0.0001

Total # pigs treated against PE	520	2845	2325	< 0.0001

	**Vaccinated**	**Non-vaccinated**	**Reduction after vaccination**	**Reduction in %**

Total amount of oxytetracykline	8.3 kg	38.9 kg	30.5 kg	79

^a^: Chi-square test

Ten batches of pigs received treatment for post weaning diarrhoea (5 vaccinated and 5 non-vaccinated batches). Significantly fewer vaccinated pigs (N = 3274) than non-vaccinated controls (N = 3734) were treated for post weaning diarrhoea (*P *< 0.0001). The 5 vaccinated batches received sulpha/TMP treatment, but the local veterinarian instructed the farmer to use aminoglycoside treatment for the non-vaccinated batches because the diarrhoea in these pigs theoretically could be caused by *L. intracellularis*. Therefore, 4 out of the 5 non-vaccinated batches were treated with sulpha/TMP. The use of different products made a comparison between the total amount of antibiotic used for vaccinated and non-vaccinated batches impossible.

In the fattening units, only one batch of pigs (vaccinated) received group medication. This batch was treated with Tilmicosin (Pulmotil, Elanco Animal Health, Lyngby, Denmark) because the pigs suffered from a lower respiratory tract infection.

Slaughterhouse data were available from 3471 pigs (2083 vaccinated and 1388 non-vaccinated). These pigs originated from the last 10 batches. 2347 pigs (1524 vaccinated and 823 non-vaccinated) were fattened at one of the fattening sites associated with the sow herd, whereas 1124 pigs (559 vaccinated and 565 non-vaccinated) were sold before fattening. Vaccination improved the ADWG from weaning to slaughter by 46 g/day (*P *= 9.55 × 10^-31^), shortened the period from weaning to slaughter by 8 days (*P *= 2.01 × 10^-45^) and increased the carcase weight by 1.25 kg (*P *= 4.54 × 10^-05^).

The study focused on oral (group) medication rather than parenteral treatment as oral medication was believed to contribute the most to the overall consumption of antibiotics. Vaccination significantly reduced the number of pigs treated with oxytetracykline, but a significant reduction was not detected for the number of batches treated. A data set like the present with a high number of un-treated batches gives rise to a high number of ties in a non-parametric statistical analysis. Therefore, it was actually the low baseline level of consumption of antibiotics that prevented significant differences to be obtained even though the total consumption was reduced by as much as 79%.

The antibiotics for treatment of post weaning diarrhoea were used equally for vaccinated and non-vaccinated batches of pigs, but at the individual level, a significant reduction in the number of treated pigs was observed after vaccination. The live oral vaccine for prevention of PE might have a stabilizing effect on the gut flora thereby also reducing the need for treatment against post weaning diarrhoea.

The reduced use of antibiotics in the nursery did not affect the production parameters negatively. On the contrary, the vaccinated pigs showed highly significant improvements. Vaccination against *L. intracellularis *probably protects the pigs even before onset of disease, thus preventing damage to the intestinal mucosa. The improved growth rate of the vaccinated pigs might indicate an advantage of prophylaxis compared to treatment, i.e. due to prevention of chronic lesions or depressed growth during episodes of diarrhoea

## Abbreviations

ADWG: Average daily weight gain; SPF: Specific pathogen free; This term refers to absence of infection with *Mycoplasma hyopneumoniae*, *Actinobacillus pleuropneumoniae *(serotypes 1–10 and 12), *Brachyspira hyodysenteriae*, toxin producing *Pasteurella multocida*, *Sarcoptes Scabiei *var. *suis*, *Haematopinus suis *and Porcine Reproductive and Respiratory Syndrome virus (all subtypes). The herd was also considered free of a number of infectious diseases according to the national disease status [9].

## Competing interests

Both authors are employed by Boehringer-Ingelheim AS, the producer of the vaccine that was tested. Still, the authors approached the study and the data scientifically and did not in any way distort data to obtain results in favour of the vaccine.

## Authors' contributions

PHR conceived of the study and participated in its design and coordination. HB performed the statistical analysis and drafted the manuscript. Both authors read and approved the final manuscript.
